# Ontology characterization, enrichment analysis, and similarity calculation‐based evaluation of disease–syndrome–formula associations by applying SoFDA

**DOI:** 10.1002/imt2.80

**Published:** 2023-01-10

**Authors:** Yudong Liu, Jia Xu, Zecong Yu, Tong Chen, Ning Wang, Xia Du, Ping Wang, Xuezhong Zhou, Haiyu Xu, Yanqiong Zhang

**Affiliations:** ^1^ Institute of Chinese Materia Medica China Academy of Chinese Medical Sciences Beijing China; ^2^ Guang'anmen Hospital China Academy of Chinese Medical Sciences Beijing China; ^3^ Institute of Medical Intelligence and Beijing Key Lab of Traffic Data Analysis and Mining, School of Computer and Information Technology Beijing Jiaotong University Beijing China; ^4^ National Resource Center for Chinese Materia Medica Chinese Academy of Chinese Medical Sciences Beijing China

**Keywords:** clinical symptom‐based diagnosis and therapy, disease–syndrome–formula association, heterogeneous biomedical network, precision medicine, similarity calculation‐based association evaluation, traditional Chinese medicine syndrome

## Abstract

Clinical symptom‐based diagnosis and therapy play a crucial role in personalized medicine and drug discovery. The syndromes, distinctive groups of clinical symptoms summarized by traditional Chinese medicine (TCM) theories and clinical experiences, are used as the core diagnostic criteria and therapeutic guidance in TCM. However, there is still a lack of standardized data, information, and intrinsic molecular basis to help TCM syndromes better classify diseases and guide tailored medications. To address this problem, we built the first integrated web platform, SoFDA (http://www.tcmip.cn/Syndrome/front/), with a curated ontology of 319 TCM syndromes, 8045 diseases, and 1359 TCM herbal formulas and their relationships with genes, diseases, and formulas. This platform proposed an association measurement by calculating Jaccard/Cosine similarities between TCM syndromes and their related biomedical entities with case and control validations. On this basis, the SoFDA platform enables biomedical and pharmaceutical scientists to rank and filter the most promising associations for disease diagnosis and tailored interventions. Conversely, the targeted gene sets and symptom sets can also be associated with TCM syndromes, formulas, and diseases for function illustration. Notably, SoFDA explores the multi‐way associations among diseases, TCM syndromes, symptom genes, herbal formulas, drug targets, and pathways in heterogeneous biomedical networks with lots of customization. The protocol here implements all the analyses above using the SoFDA platform. Collectively, SoFDA may provide insights into the biological basis of disease‐specific TCM syndromes and the underlying molecular mechanisms, as well as a tailored treatment for single or multiple symptoms within a syndrome.

## Highlights

- SoFDA is the first manually curated public traditional Chinese medicine (TCM) syndrome annotation database.
- SoFDA explores the multiway associations among diseases, symptoms, genes, herbal formulas, drug targets, and pathways in heterogeneous biomedical networks with lots of customizations.
- A similarity‐based association measurement was used to evaluate the association levels of disease–TCM syndrome, TCM syndrome–TCM syndrome and formula–TCM syndrome, and disease–TCM syndrome–formula by calculating the Jaccard/Cosine similarities.

## Graphical abstract

SoFDA is the first integrated web platform, SoFDA (http://www.tcmip.cn/Syndrome/front/), with a curated ontology of 319 traditional Chinese medicine syndromes and their relationships with genes, diseases, and formulas. This platform enables biomedical and pharmaceutical scientists to rank and filter the most promising associations for disease diagnosis and tailored interventions.

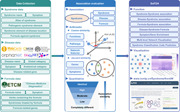

## INTRODUCTION

Precision medicine and symptom management are prominent topics in the field of symptom science research. Accumulating studies have revealed that clinical symptoms are essential for both drug discovery and customized treatment. Development and progression of most diseases have been indicated to be associated with the advent of a group of clinical symptoms at the same time, suggesting that disease subtypes should be classified based on clinical symptom groups rather than a single symptom. Similar to multitarget medications, symptom‐based diagnosis may assist physicians in correctly identifying disease subtypes and fostering the discovery of therapeutic combinations [1, 2, 3].

As the core diagnostic and therapeutic criteria in traditional Chinese medicine (TCM), syndromes are distinctive groups of clinical symptoms that have been condensed based on thousands of years of clinical experience in traditional medical interventions [4]. TCM syndrome is a kind of distinct diagnosis derived from TCM theories and clinical skills that are solely based on the clinical manifestations (i.e., symptoms and signs) of patients [5]. In the practical TCM clinical setting, both TCM syndromes and modern disease diagnoses may be given to each patient. For example, for a patient with coronary heart disease and the manifestations of a dark red tongue, the corresponding TCM diagnosis may be "blood stasis syndrome." Therefore, TCM syndromes and modern medicine (MM) diseases together propose a paralleling schema of diagnoses in TCM clinical settings, implying that biomedical associations may exist between some specific TCM syndromes and MM diseases. TCM syndromes specific to the disease may help to narrow the differential diagnosis, allow for more individualized management, and improve patients' prognoses [6]. The associations between syndromes, diseases, and formulas may be indirect or casual, and play an important role in meeting medical needs, since they may vary in different disease statuses with clinical symptoms and signs [7].

When the 11th iteration of the International Statistical Classification of Diseases and Related Health Problems (ICD‐11) was adopted in 2019 [8], TCM was officially added as a brand‐new chapter on traditional medicine conditions, indicating that syndrome‐based diagnosis in TCM has been accepted by modern healthcare systems. TCM has played a special and important role in the prevention and treatment of COVID‐19, and many characteristic databases have also been established during this period [9, 10]. As a bridge to MM, more attention needs to be given to developing the standardization of TCM syndromes and the consistency of TCM nomenclature [11]. The widespread adoption and continued development of TCM syndromes in the global medical system are constrained by the absence of precise knowledge of a molecular basis. Therefore, we developed the SoFDA platform, the first manually curated public TCM syndrome annotation database [12]. SoFDA contains a database of syndrome ontology with rationality and provides an authoritative platform for in‐depth research on the molecular mechanism of the TCM syndrome and the associations among diseases, TCM syndromes, and herbal formulas. Thus, SoFDA provides the evaluation results of TCM syndrome–TCM syndrome, TCM syndrome–disease, and disease–TCM syndrome–TCM formula associations, giving tremendously valuable guidance for accurate clinical diagnosis, designing the appropriate treatment, and drug discovery programs.

## DATA SUPPORT AND SOURCES

The SoFDA platform integrates data from multiple sources and offers exhaustive details on the most prevalent TCM syndromes, as well as the associated diseases and the corresponding TCM formulas. Users can interact with collaborative data analysis using TCM syndrome‐, disease‐, and TCM formula‐related data.

### TCM syndrome ontology

**Figure 1:**
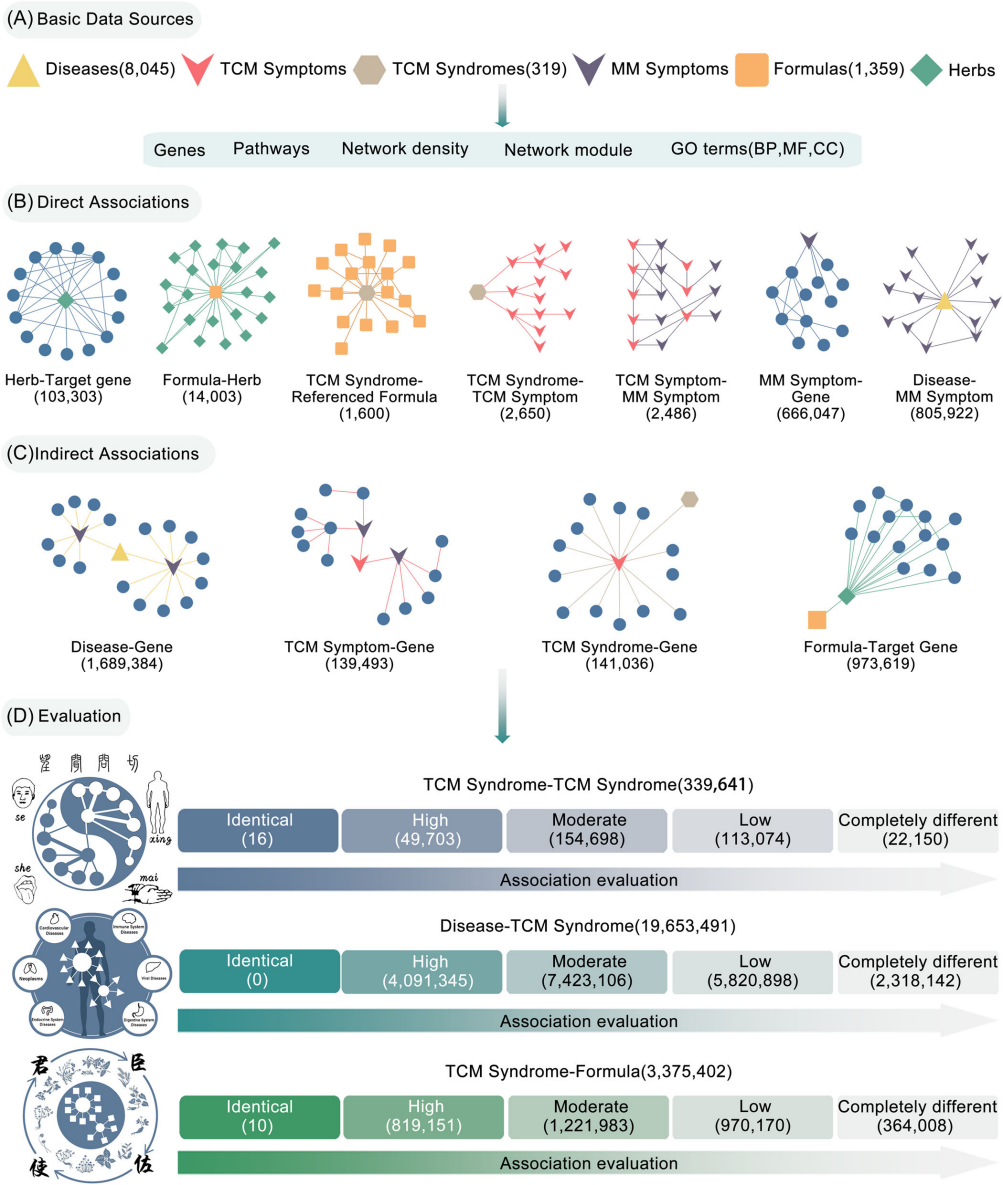
Data support and sources of the SoFDA platform. (A) Basic data sources. (B) Direct associations. (C) Indirect associations. (D) Association evaluation in the SoFDA platform. BP, biological process; CC, cellular component; GO, Gene Ontology; MF, molecular function; MM, modern medicine; TCM, traditional Chinese medicine.

The SoFDA platform provides detailed information on 319 TCM syndromes (including 9 TCM syndromes of COVID‐19), containing syndrome name, alias of syndrome, symptoms, syndrome element of disease location, pathogenic syndrome element, and formula against syndrome, which were, respectively, collected from the monograph of "Chinese Medicine Diagnostics" and "Treatment Plan of Traditional Chinese Medicine for COVID‐19 (Trial Eighth Edition)". Of note, the category and code information of each TCM syndrome were collected from "Classification and codes of diseases and ZHENG of TCM (GB/T 15657‐1995, released in 1995 and 2020)" and ICD‐11. Owing to the known limitations in completeness and quality of currently available data on clinical manifestations and intrinsic mechanisms of TCM syndromes, we here first confirmed the links between TCM symptoms and MM symptoms by clinical expert consensus and subsequent manual verification. Importantly, 319 TCM syndromes and the related 1610 TCM symptoms are indirectly associated with 3955 genes using the intermediate relationships between TCM symptoms and MM symptoms manually (Figure 1A).

### Disease‐related data

The SoFDA platform provides detailed information on 8045 human diseases, including disease names, global categories, anatomical categories, symptoms, disease‐related genes, and hallmark gene set annotations. All information was collected from the human disease database [13] (MalaCards v5.0, https://www.malacards.org/), GeneCards [14] (GeneCards v5.13, https://www.genecards.org/), Human Phenotype Ontology [15] (HPO, Released in 2018, https://hpo.jax.org/app/), Online Mendelian Inheritance in Man [16] (OMIM, Released in April 2018, https://omim.org/), database of gene–disease associations [17] (DisGeNET v5.0, https://www.disgenet.org/), and the portal for rare diseases and orphan drugs [18] (ORPHANET v5.49.0, https://www.orpha.net/consor/cgi-bin/index.php). The disease section also contains comprehensive data on 8937 MM symptoms, 10273 related genes, and their associations, including 1,689,384 associations among diseases and genes, 805,922 associations involving diseases and MM symptoms, and 666,074 associations between MM symptoms and genes (Figure 1A).

### TCM formula‐related data

The SoFDA platform provides detailed information on 1359 TCM formulas, collected from the monograph of "Chinese Medicine Diagnostics" and the Encyclopedia of Traditional Chinese Medicine (ETCM) database [19] (the ETCM, http://www.tcmip.cn/ETCM/index.php/Home/Index/), including the name of the formula, herbal composition, symptoms, and TCM syndromes intervening by TCM formulas. A total of 1214 herbs and 1796 putative target genes of these TCM formulas are also provided based on the data collected from the ETCM database. Moreover, there are 14,003 TCM formula–herb and 103,303 herb–target gene direct associations, respectively, as well as 973,619 indirect associations between TCM formulas and putative target genes (Figure 1A).

## ASSOCIATION EVALUATION

### Direct associations

The SoFDA platform provides seven direct associations of disease–MM symptom, MM symptom‐gene, TCM syndrome–TCM symptom, TCM syndrome‐referenced formula, TCM symptom–MM symptom, formula–herb, and herb–target gene with the assistance of fundamental data. Notably, the TCM symptoms within TCM syndromes are precisely matched to MM symptoms via clinical expert consensus and subsequent manual verification. The formula–herb and the herb–target gene associations were both obtained from the ETCM database (Figure 1B).

### Indirect associations

SoFDA offers indirect associations of eight nonadjacent characteristics in addition to the aforementioned seven direct associations utilizing nearby feature parameters, such as disease–gene, TCM symptom–gene, TCM syndrome–gene, formula–target gene, disease–TCM syndrome, TCM syndrome–TCM syndrome, formula–TCM syndrome, and disease–TCM syndrome–formula associations. The indirect associations between diseases and genes, as well as between TCM Symptoms and genes, are both obtained using MM symptoms as middle parameters. According to the TCM symptom–gene relationship, TCM syndromes are linked with genes. Meanwhile, the indirect associations of formula–target gene are inferred through herbs (Figure 1C).

## THE FUNCTIONALITY OF THE SoFDA PLATFORM

SoFDA can evaluate the indirect associations of disease–TCM syndrome, TCM syndrome–TCM syndrome, formula–TCM syndrome, and disease–TCM syndrome–formula by calculating Jaccard similarities and cosine similarities of six characteristic parameters, including symptoms (syndrome‐related TCM symptoms and disease‐related MM symptoms), genes (symptom‐related genes and putative target genes of TCM herbal formulas), and the enriched functional terms based on Gene Ontology (GO) and Reactome pathways, network modules, and network density.

Enrichment analysis using the state‐of‐the‐art overlapping community discovery approach BigClam yields the network modules for syndromes, diseases, and formulae [20]. Network density, a concept used to characterize the density of edges between nodes in a network, was utilized to examine the association degree of the network, and the definition is as follows:

$$d\left(G\right)=\frac{2L}{N\left(N-1\right)}$$

where d(G) is the network density, and *L* represents the number of edges in the network, and *N* represents the number of nodes in the network.

In addition, a quartile categorization approach was used to evaluate each indirect association level. Specifically, the similarity values were sorted in descending order and the first quantile (Q1) and the third quantile (Q3) of the sequence were calculated, respectively. Taking these two indicators as the grading standard, all the indirect association levels were divided into five levels.

(1) Identical (the similarity values are 1.0).
(2) High (the similarity values are greater than or equal to Q1).
(3) Moderate (the similarity values are between Q1 and Q3).
(4) Low (the similarity values are lower than the Q3).
(5) Completely different (the similarity values are 0) levels.

The associated methodologies' accuracy has been confirmed in our previous study [12]. SoFDA offers association evaluations in several circumstances.

### Syndrome–syndrome association

Syndrome differentiation is the core of TCM diagnosis and the foundation of TCM therapy. Accurate syndrome differentiation may be of great significance to ensure the rational treatment of diseases. SoFDA can assess the connection between various symptoms according to the clinical symptoms and the related genes, the enriched GO items, pathways, network density, and network modules to identify the commonness and individuality of similar/different syndromes (Figure 1D).

### Disease–syndrome association

The theory of TCM follows the principle of syndrome differentiation and treatment to determine the clinical characteristics of diseases. There is no independent "one‐to‐one correspondence" between diseases and syndromes; that is, different diseases may have the same syndrome, and the same disease may have different syndromes, which formed the principle of "Treating different diseases in the same way" and "Treating the same disease in different ways," respectively. On this basis, SoFDA can evaluate the associations between diseases and syndromes from a molecular perspective, which may be of great significance to clarify the biological basis of the principles of "disease and syndrome combination" in TCM and to standardize the future research of TCM syndrome (Figure 1D).

### Formula–syndrome association

"Formulas with the correspondence to TCM Syndromes," the notion of healing developed during the drawn‐out process of treating diseases with prescription syndromes, is the integration of syndrome‐differentiation procedures and concepts utilized in TCM clinics. As a result, the evaluation of the associations between TCM syndrome and TCM formula may assist in the compatibility of syndrome differentiation, improve clinical efficacy, and increase the variety of pharmacological uses in clinical settings. The terms "syndrome" and "formula" can be linked based on similarities in clinical symptoms, signs, chemical indicators of TCM syndromes and indications of TCM formulas, syndrome genes and formula targets, and biological functions and pathways (Figure 1D).

### Disease–syndrome–formula association

**Figure 2:**
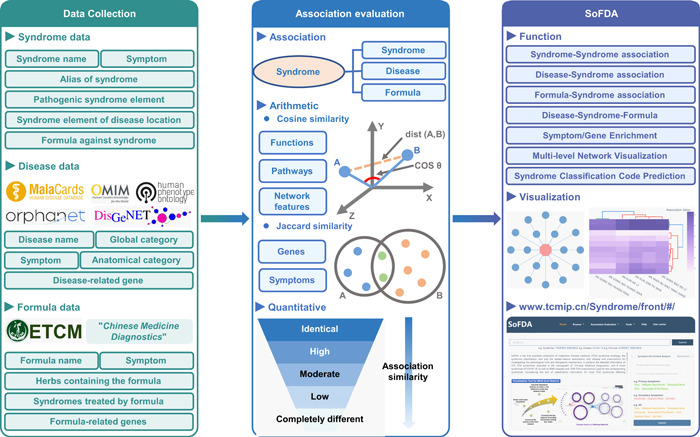
Protocols for the data collection, the association evaluation, and the overview of the SoFDA platform. ETCM, Encyclopedia of Traditional Chinese Medicine.

"Disease and syndrome combination" and "Formulas corresponding to the syndromes" are the essence of TCM theory. Clinical observations show that all diseases may be caused by an imbalance in the patient's body, which refers to the syndrome. All the therapies and formulas in TCM are carried out based on the patients' syndrome situation. To quantitatively evaluate the association of syndrome, disease, and formula, we here first identify the syndrome features for further stratification of the patients' conditions with a certain disease, which may help the improvement of the efficacy of the selected intervention. After that, a quantitative evaluation of the correspondence of formulas to the combination of disease and syndrome is performed according to the symptoms, targeting pathological change‐related genes, functions, and pathways, which may be beneficial to reveal the complex scientific connotation of formulae based on "Disease‐TCM Syndrome‐TCM Formula Association." This function also provides guidance to the clinical practice, presents a new strategy for the research on "treating the same disease with different formulas" and "treating different diseases with the same formula" theories, and notably, makes a difference to the innovation development of basic theories of TCM, as well as the research and production of modern Chinese drugs. According to the clinical symptoms and the related genes, enriched GO items, pathways, network densities, and network modules, SoFDA evaluates the disease–TCM syndrome–TCM formula association with the two selections—"Disease‐TCM Syndrome‐TCM Formula Association with the Same Disease" and "Disease‐TCM Formula Association with the Same Syndrome" (Figure 2).

### Symptom/gene enrichment analysis

The "Symptom Enrichment Analysis" and "Gene Enrichment Analysis" tools of SoFDA facilitate users in obtaining the significantly associated TCM syndromes of a given symptom list and the TCM/MM symptoms, TCM syndromes, TCM formulas, functional GO items, and pathways involved by a given gene list, respectively (Figure 2).

### Multilevel network visualization

SoFDA provides a program for a multilevel network of disease‐syndrome–formula visualization, which can be used to illustrate the associations around diseases, TCM syndromes, symptom genes, herbal formulas, drug targets, and pathways according to users' designation and modification. This tool is intended to make it simpler for individuals to understand diseases, TCM syndromes, and TCM herbal formulas from a molecular scientific viewpoint. Additionally included in SoFDA are the gene–gene interaction network visualization and network module statistics, as well as GO terms or pathways that are enriched by genes associated with certain diseases and TCM syndromes, or which are targeted by TCM herbal formulas (Figure 2).

### Syndrome classification code prediction

SoFDA supplies a prediction tool for the potential candidate classification and coding of TCM syndromes, which was established by an XGBoost model based on the clinical symptoms of TCM syndromes, syndrome‐related genes, GO items, pathways, and gene–gene interaction network modules. We also evaluated the effectiveness of this prediction tool using the accuracy, precision, recall, and F1 scores as indicators, and the results were 92.42%, 89.88%, 77.00%, and 82.92%, respectively.

## IMPLEMENTATION OF A WEB SERVER

SoFDA was constructed with a front‐end and back‐end separation framework. A MySQL database was used to store data that had been manually vetted and computationally processed. The immensely effective Javascript framework Vue.js was used to create the front‐end framework. Data visualization was accomplished using the javascript libraries ECharts and plotly. The high‐level Python web framework Django was used as the back‐end for data extracting and data processing. An Apache server serves as the website's host. For a variety of compatibility, the SoFDA website was tested using Google Chrome, Mozilla Firefox, Opera, and Safari.

## USE PROCEDURES

### Web navigation

Users of SoFDA can browse, search, analyze, and download data on diseases, TCM syndromes, and TCM formulas, as well as carry out visual analyses using the logical interactive interface.

### Home page

**Figure 3:**
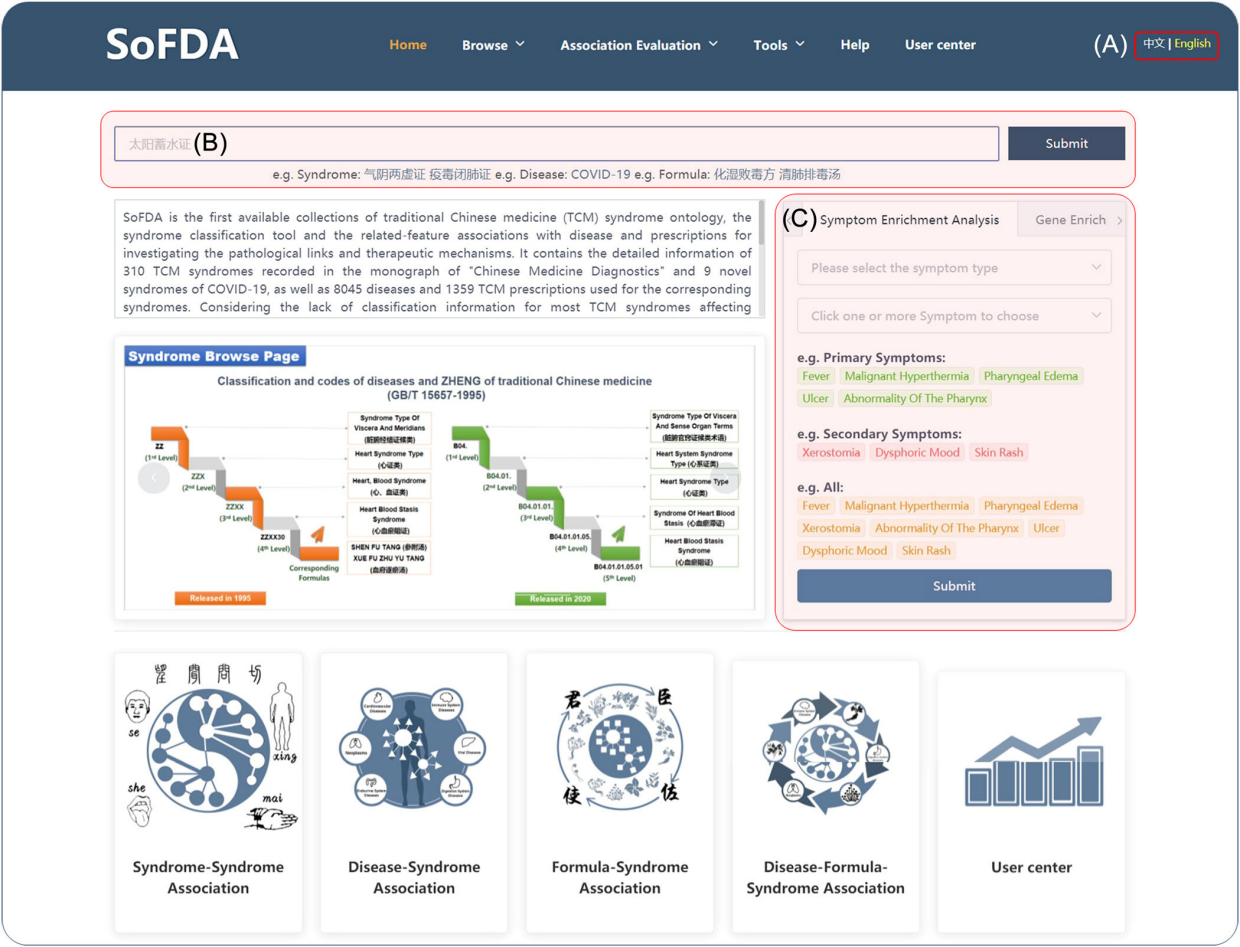
The home page of the SoFDA platform.

SoFDA has two interfaces for both the English and Simplified Chinese versions, and users may freely switch them on the right side of the top of each webpage (Figure 3A). Users can enter any phrase contained in a disease, TCM syndrome, TCM herbal formula, symptom, or gene name in the search bar to conduct a global search in SoFDA (Figure 3B). Additionally available on the home page are the "Gene Enrichment Analysis" and "Symptom Enrichment Analysis" tools (Figure 3C).

### Symptom enrichment analysis

Users first select the Primary Symptom, Secondary Symptom, or Primary & Secondary Symptom options (Supporting Information: Figure S1B), and then select one or more symptoms as needed (Supporting Information: Figure S1C). After that, click "Submit" to check the enrichment analysis results.

### Gene enrichment analysis

Users first select TCM syndromes, TCM symptoms, MM symptoms, TCM formulas, biological processes, cellular components, molecular functions, and Reactome pathways as needed (Supporting Information: Figure S1E) and then paste a gene list (Supporting Information: Figure S1F). After that, click "Submit" to check the enrichment analysis results.

### Browse

#### Syndrome browse

**Figure 4:**
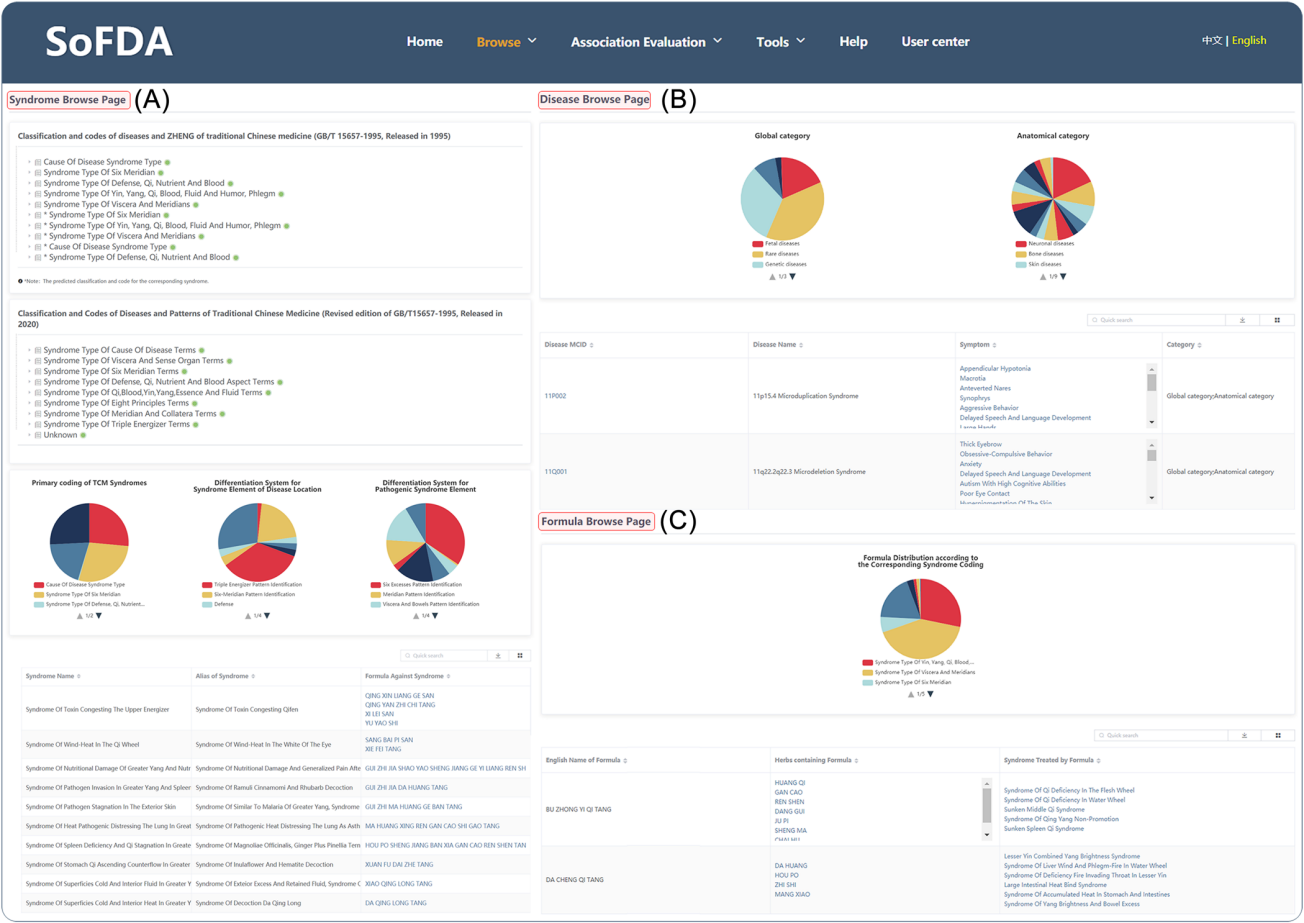
A practical guide to browse the SoFDA platform (merge page). (A) Syndrome page. (B) Disease page. (C) Formula page.

SoFDA provides two different classification and code categories of TCM syndromes, including "Classification and codes of diseases and ZHENG of TCM (GB/T 15657‐1995, released in 1995)" and "Classification and Codes of Diseases and Patterns of Traditional Chinese Medicine (Revised edition of GB/T 15657‐1995, released in 2020)," in addition to a brief table of the "syndrome name" and "formula against syndrome (Figure 4A). Clicking on the "syndrome ID" will take users to the details page, which can display syndrome name, alias of syndrome, symptoms, syndrome element of disease location, pathogenic syndrome element, symptom‐related genes, and formulas against syndrome (Supporting Information: Figure S2A).

SoFDA can perform enrichment analysis and visualization from five modules in this browse, including functional enrichment of syndrome‐related genes, interaction network of syndrome‐related genes, top 10 network modules of syndrome‐related genes, functional enrichment of network modules, and associations with other syndromes, by using syndrome‐related genes as intermediaries (Supporting Information: Figure S3A–E).

#### Disease browse

All types of diseases are categorized by Anatomical category and global category in the human disease database (MalaCards v5.0, https://www.malacards.org/). Users are initially shown a brief table of the "Disease MCID," "Disease name," and "Symptom" (Figure 4B). Clicking on the "Disease MCID" will take users to the detailed information page, displaying disease name, category, symptom, CrossRef, and disease‐related genes (Supporting Information: Figure S2B).

SoFDA can perform enrichment analysis and visualization from five modules in this browse page, including functional enrichment of disease‐related genes, interaction network of disease‐related genes, top 10 network modules of disease‐related genes, functional enrichment of network modules, and associations with other syndromes, by using disease‐related genes as intermediaries (Supporting Information: Figure S3A–E).

#### Formula browse

A brief table of the "English name of formula," "Herbs containing formula," and "Syndrome treated by Formula" is initially shown on the browse page of TCM formulas (Figure 4C). Clicking on the "Formula ID" will take users to the detailed information page, displaying disease name, category, symptom, CrossRef, and disease‐related genes (Supporting Information: Figure S2C).

SoFDA can perform enrichment analysis and visualization from five modules, including functional enrichment of formula‐related genes, interaction network of formula‐related genes, top 10 network modules of formula‐related genes, functional enrichment of network modules, and associations with other syndromes, by using disease‐related genes as intermediaries (Supporting Information: Figure S3A–E).

### Association evaluation

#### Syndrome–syndrome association

**Figure 5:**
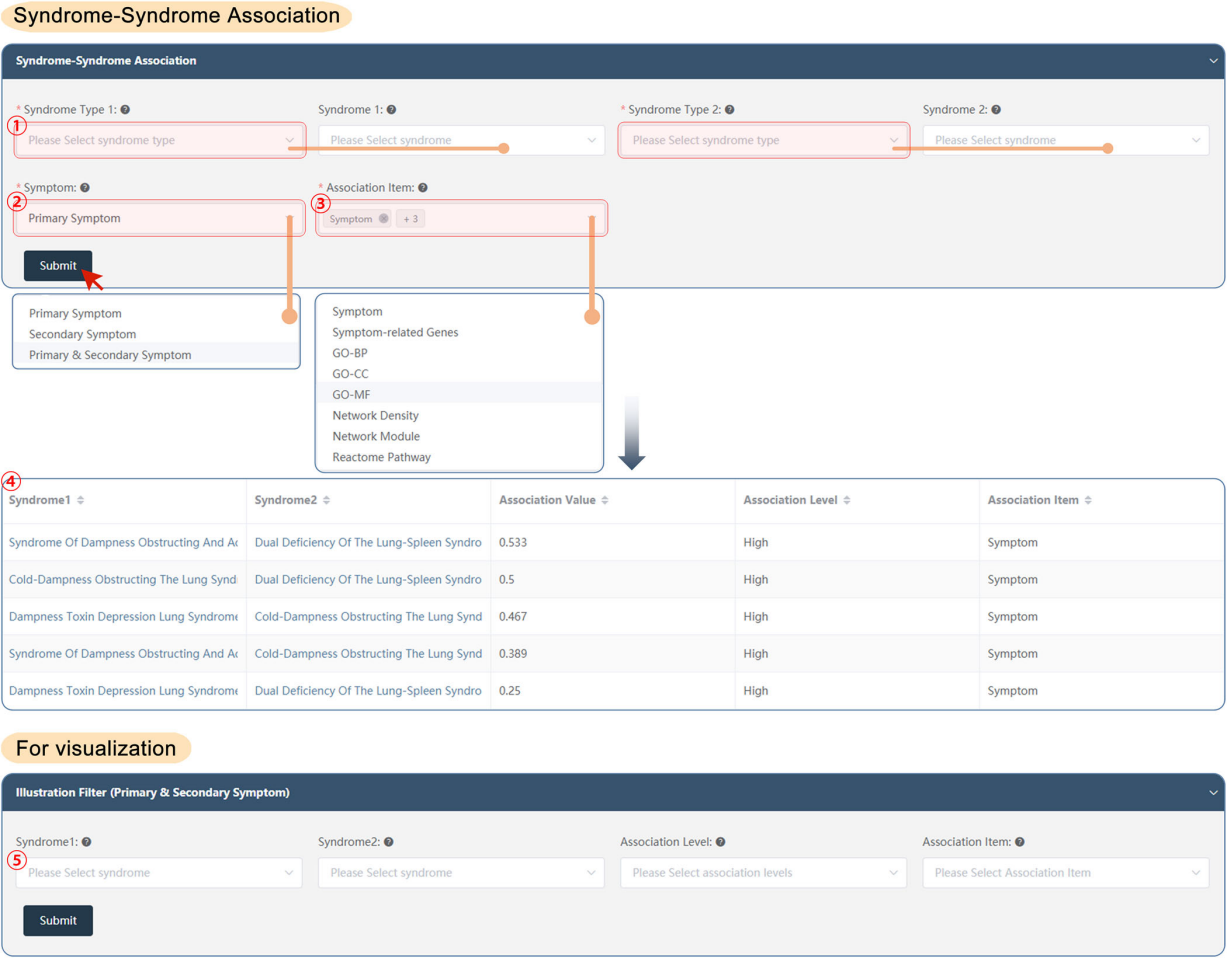
The syndrome–syndrome association of the SoFDA platform. BP, biological process; CC, cellular component; GO, Gene Ontology; MF, molecular function.

SoFDA evaluates the associations among different TCM syndromes according to the clinical symptoms and the related genes, the enriched GO items, pathways, network density, and network modules based on the following steps:

(1) Select the syndrome Type 1, Type 2, and the corresponding syndromes as needed to evaluate the associations (every item allows for multiple selections).
(2) Select the symptom types: Primary, secondary, or primary and secondary symptoms.
(3) Select the association types: About the clinical symptoms and the related genes, the enriched GO items, pathways, network density, and network modules as needed (allows for multiple selections).
(4) Submit: The results will show the association value, association level, and association item between Syndrome 1 and Syndrome 2 (Figure 5).
  If needed visualization
(5) Please select the syndromes once more, as well as the association levels and items that are needed to illustrate the association evaluation results, and then SoFDA may show the syndrome‐syndrome associations in the forms of various graphics, such as heatmap, network, or scatter (Supporting Information: Figure S4A–C).

#### Disease–syndrome association

**Figure 6:**
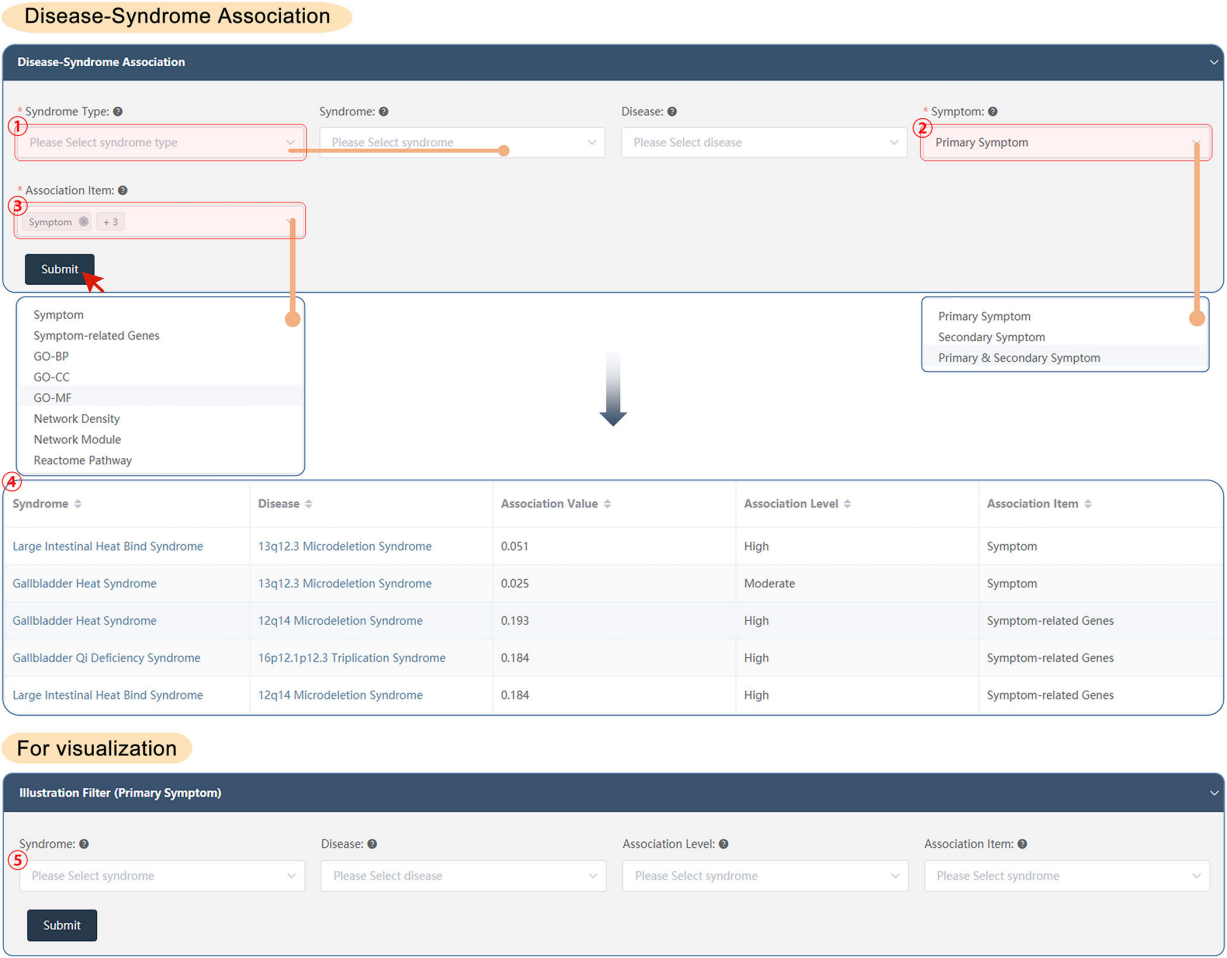
The disease–syndrome association of the SoFDA platform. BP, biological process; CC, cellular component; GO, Gene Ontology; MF, molecular function.

SoFDA evaluates the associations among syndromes and diseases according to the clinical symptoms and the related genes, the enriched GO items, pathways, network density, and network modules based on the following steps:

(1) Select the syndrome types, the corresponding syndromes, and the diseases as needed to evaluate the associations (every item allows for multiple selections).
(2) Select the symptom types: Primary, secondary, or primary and secondary symptoms.
(3) Select the association types: About the clinical symptoms and the related genes, the enriched GO items, pathways, network density, and network modules as needed (allows for multiple selections).
(4) Submit: The results will show the association value, association level, and association item between syndrome and disease (Figure 6).

If needed visualization:

(5) Please select the syndromes and diseases once more, as well as the association levels and items that are needed to illustrate the association evaluation results, and then SoFDA may show you the syndrome‐syndrome associations in the form of various graphics, such as heatmap, network, or scatter (Supporting Information: Figure S4A–C).

#### Formula–syndrome association

**Figure 7:**
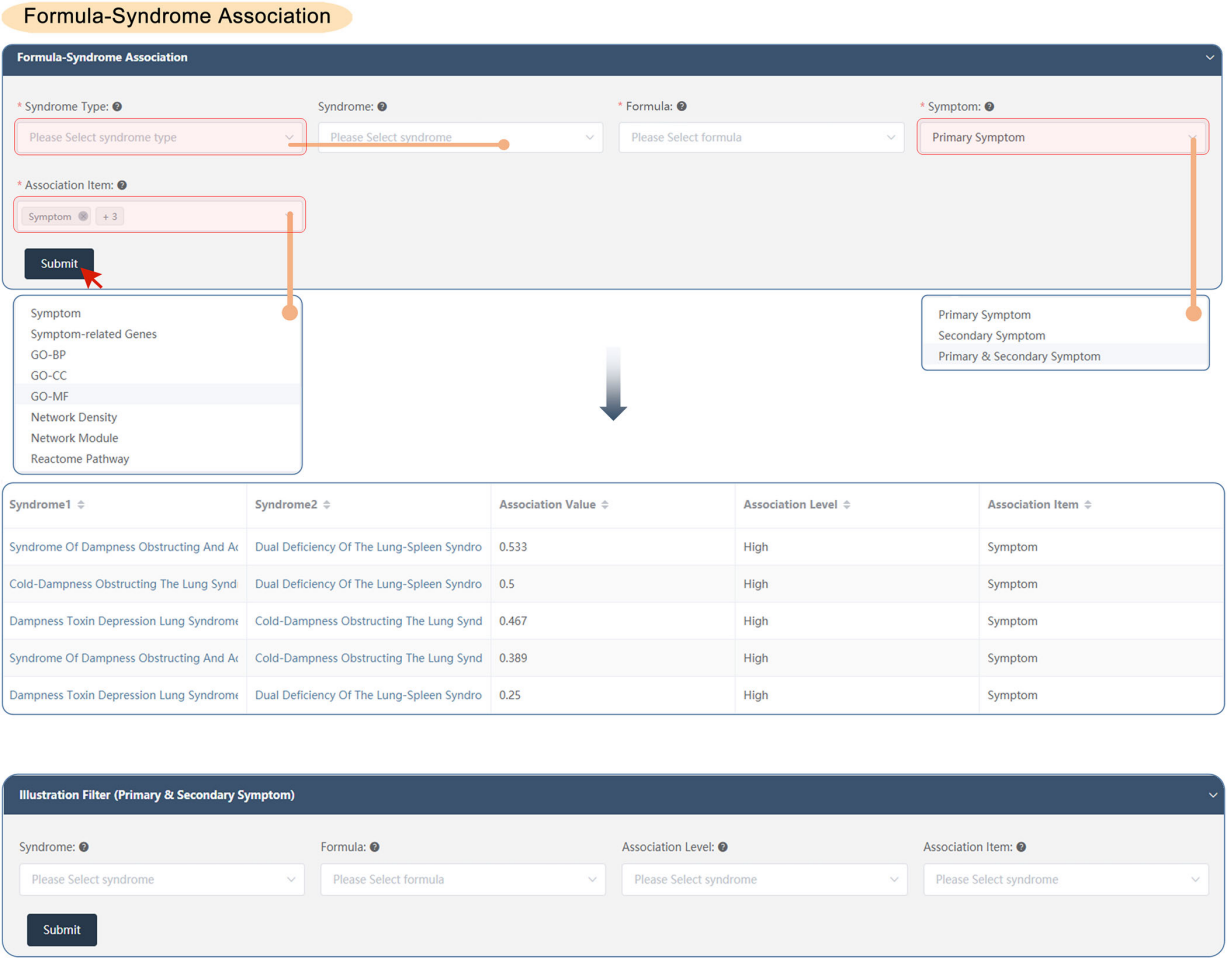
The formula–syndrome association of the SoFDA platform. BP, biological process; CC, cellular component; GO, Gene Ontology; MF, molecular function.

SoFDA evaluates the associations among syndromes and formulas according to the clinical symptoms and the related genes, the enriched GO items, pathways, network density, and network modules based on the following steps:

(1) Select the syndrome types, the corresponding syndromes, and the formulas as needed to evaluate the associations (every item allows for multiple selections).
(2) Select the symptom types: Primary, secondary, or primary and secondary symptoms.
(3) Select the association types: About the clinical symptoms and the related genes, the enriched GO items, pathways, network density, and network modules as needed (allows for multiple selections).
(4) Submit: The results will show the association value, association level, and association item between the syndrome and formula (Figure 7).

If needed visualization:

(5) Please select the syndromes and formula once more, as well as the association levels and items that are needed to illustrate the association evaluation results, and then SoFDA may show you the syndrome‐syndrome associations in the form of various graphics, such as heatmap, network, or scatter (Supporting Information: Figure S4A–C).

#### Disease‐syndrome‐formula association

**Figure 8:**
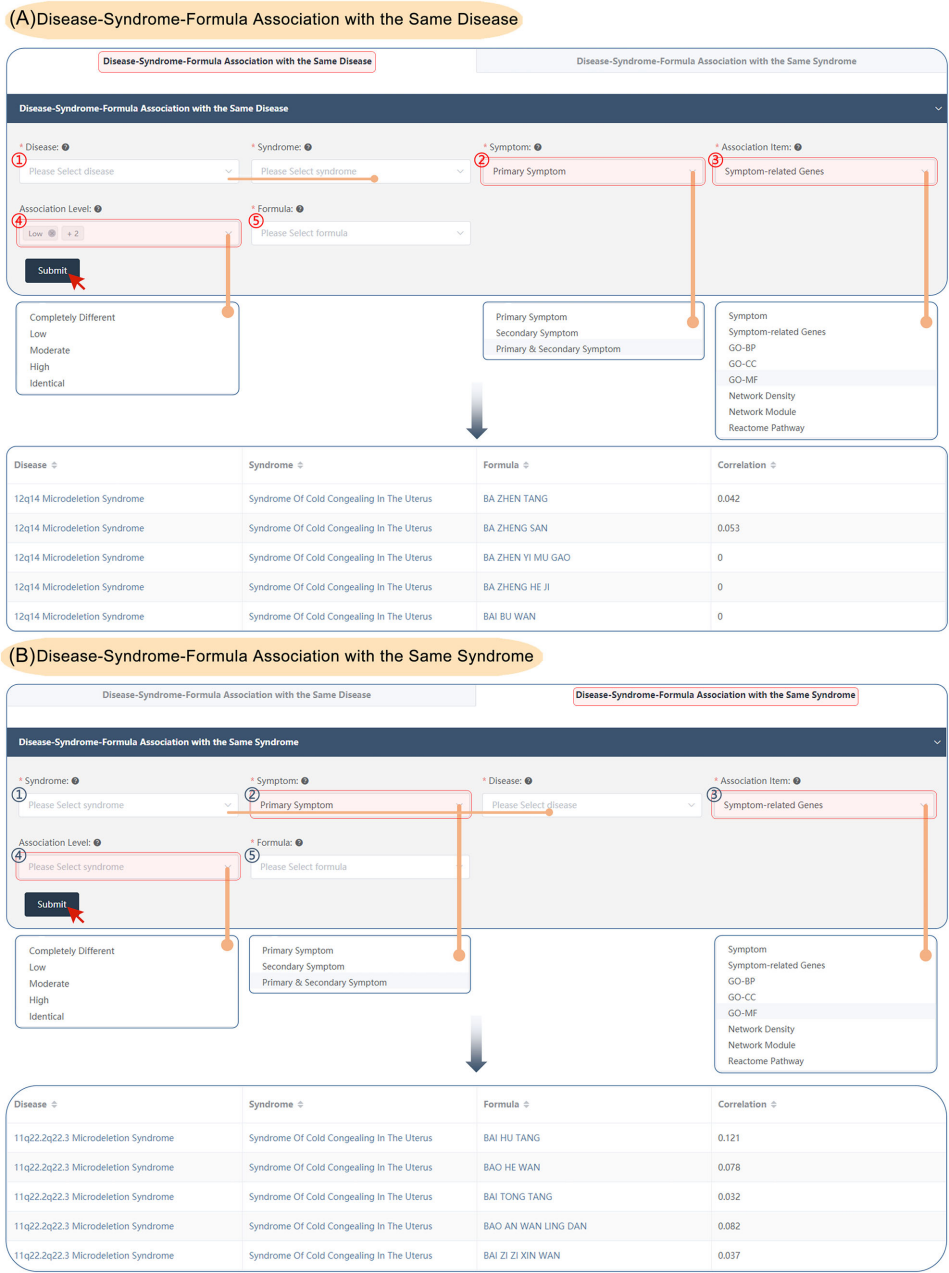
The disease–syndrome–formula association of the SoFDA platform. (A) Disease‐syndrome–formula association with the same disease. (B) Disease–syndrome–formula association with the same syndrome. BP, biological process; CC, cellular component; GO, Gene Ontology; MF, molecular function.

SoFDA evaluates the associations among syndrome‐disease‐formula association with the two selections—"Syndrome‐Disease‐Formula Association with the Same Disease" and "Syndrome‐Disease‐Formula Association Same Syndrome" according to the clinical symptoms and the related genes, the enriched GO items, pathways, network density, and network modules based on the following steps:

Disease–syndrome–formula association with the same disease:

(1) Select the disease and syndromes (allows for multiple selections) as needed to evaluate the associations.
(2) Select the symptom types: Primary, secondary, or primary and secondary symptoms.
(3) Select the association types: About the clinical symptoms and the related genes, the enriched GO items, pathways, network density, and network modules as needed.
(4) Select the association levels of the syndrome‐disease association: Identical, high, moderate, low, and completely different (allows for multiple selections).
(5) Select the formulas as needed to evaluate their associations with the syndrome–disease combination (allows for multiple selections).
(6) Submit: The results will immediately visualize the “Disease‐Syndrome‐Formula Association with the Same Disease” page as well as the correlation of disease–syndrome–formula (Figure 8A).

Disease–syndrome–formula association with the same syndrome:

(1) Select the syndromes and disease (allows for multiple selections) as needed to evaluate the associations.
(2) Select the symptom types: Primary, secondary, or primary and secondary symptoms.
(3) Select the association types: About the clinical symptoms and the related genes, the enriched GO items, pathways, network density, and network modules as needed.
(4) Select the association levels of the syndrome‐disease association: Identical, high, moderate, low, and completely different (allows for multiple selections).
(5) Select the formulas as needed to evaluate their associations with the syndrome‐disease combination (allows for multiple selections).
(6) Submit: The results will immediately visualize the “Disease‐Syndrome‐Formula Association with the Same Syndrome” page as well as the correlation of disease–syndrome–formula (Figure 8B).

## TOOLS

### Prediction tool for syndrome classification code

This is a prediction tool for the candidate classification and codes of TCM syndromes based on the following steps (Supporting Information: Figure S5):

(1) Select the clinical symptoms of the tested syndrome.
(2) Define the name of the tested syndrome.
(3) Click "Add" to define the names of the tested syndromes and to select the clinical symptoms if there are multiple tested syndromes.
(4) Press “Submit” and check the prediction results.

### Multilevel network visualization

The “Multi‐level network visualization" tool determines and illustrates the all‐versus‐all relationships among TCM syndromes, diseases, and TCM herbal formulas (disease–TCM syndrome, TCM syndrome–TCM syndrome, TCM formula–TCM syndrome, or disease–TCM syndrome–TCM formula), and a list of tables and images are displayed according to the association evaluation results.

Users can select one or more syndromes, diseases, and formulas on this page. After that, they can specify the clinical symptom types, association items, and levels (depending on the needs). Following the click of the "Submit" button, a variety of data formats, including tables, heatmaps, networks, and upset plots, are displayed with comprehensive results on the specified association.

**Figure 9:**
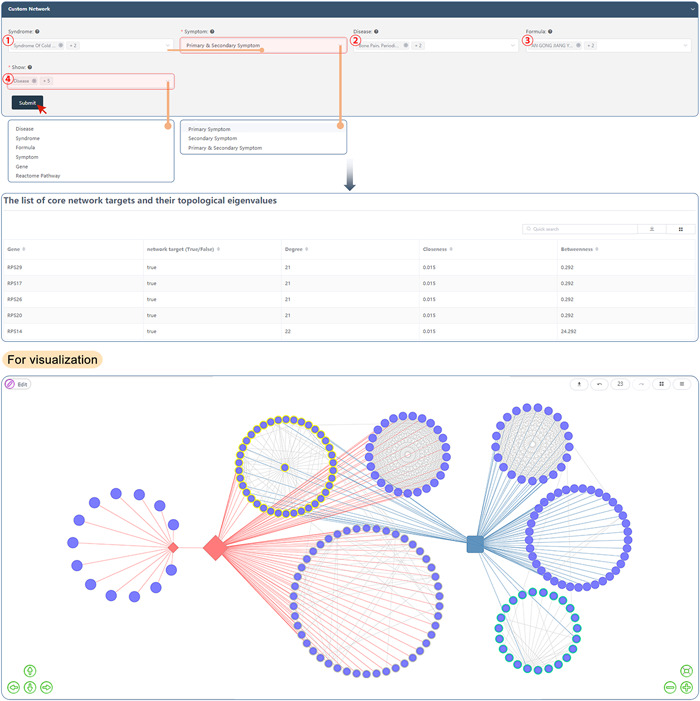
Multilevel network visualization tool of the SoFDA platform.

After clicking the "Submit" button, users can construct different kinds of multilevel networks, such as disease – TCM syndrome – pathway network, TCM herbal formula – drug target gene – pathway – TCM syndrome – disease network, and so forth. In addition, a variety of data formats, such as tables, heatmaps, networks, and upset plots, are displayed along with the comprehensive results on the specified association. Of note, users can also edit network nodes and edges as needed in this panel (Figure 9).

### Case study on COVID‐19

We herein took COVID‐19 as an example to validate the efficacy of SoFDA in improving diagnostic accuracy and therapeutic response assessments. SoFDA provides detailed information on 9 TCM syndromes of COVID‐19, and the corresponding 18 herbal formulas against these syndromes. Among them, Huashi Baidu formula (HBF) is recommended for the treatment of COVID‐19 patients with the syndrome of epidemic toxin obstructing the lung by the National Medical Products Administration. However, the molecular basis of COVID‐19 patients with the syndrome of epidemic toxin obstructing the lung and the reason for their favorable response to the treatment of HBF have not been fully elucidated. Herein, the "Disease‐Syndrome‐Formula" association was evaluated using the "Association evaluation tool" of SoFDA based on various features, including symptom‐related genes, GO terms, Reactome pathways, network modules, and network density, as the following steps: Select “Covid‐19/CVD001” as a disease;

(1) select “Syndrome of epidemic toxin obstructing the lung” as the syndrome;
(2) select “Primary & Secondary Symptom” as Symptom;
(3) select “Symptom‐related Genes,” “GO‐BP”, “GO‐CC”, “GO‐MF”, “Reactome Pathway”, “Network Module” and “Network Density” as Association Item in turn;
(4) select all association levels as association level, including “Completely Different,” “Low,” “Moderate,” “High,” and “Identical”;
(5) select “HUA SHI BAI DU FANG” as formula;
(6) click Submit button.

Following the submission, the associations among the submitted TCM syndrome, disease, and herbal formula may be obtained and output (Supporting Information: Figure S6).

## CONCLUSION

SoFDA may be a promising platform that bridges information from TCM syndromes, diseases, and TCM formulas to molecular mechanisms, which will deepen our understanding of ancient systematic medicine, TCM, and the corresponding medical intervention. Owing to the known limitations in completeness and quality of currently available data on clinical manifestations and intrinsic mechanisms of TCM syndromes, further updates of our SoFDA platform should be required if the relevant data may be standardized and available.

## AUTHOR CONTRIBUTIONS

Haiyu Xu, Yanqiong Zhang and Xuezhong Zhou conceived the study, participated in its design and coordination, and revised the manuscript. Yudong Liu drafted the manuscript. Zecong Yu, Xia Du and Ning Wang carried out the calculation and performed the statistical analyses. The other authors participated in this study. All authors read and approved the final manuscript.

## CONFLICT OF INTEREST

The authors declare no conflict of interest.

## Supporting information

Supplementary information.

## Data Availability

SoFDA is publicly accessible to all researchers (http://www.tcmip.cn/Syndrome/front/#/). Downloadable versions of all the data evaluated by SoFDA include data (.xls) and vector graphs (.svg.pdf). Please refer to the "Help" page, which includes a comprehensive user manual, for more information. Supporting Information: Materials (figures, tables, scripts, graphical abstract, slides, videos, Chinese translated version, and updated materials) may be found in the online DOI or iMeta Science http://www.imeta.science/.
