# Supplementary material for: Ontology characterization, enrichment analysis, and similarity calculation‐based evaluation of disease–syndrome–formula associations by applying SoFDA
Source: Imeta. 2023 Jan 10;2(2):e80. doi: 10.1002/imt2.80 (PMC10989962; doi:10.1002/imt2.80)
Supplement: Supplementary file 1 — Supplementary information. [file IMT2-2-e80-s001.docx]

**SUPPLEMENTARY FIGURE LEGENDS:**

**
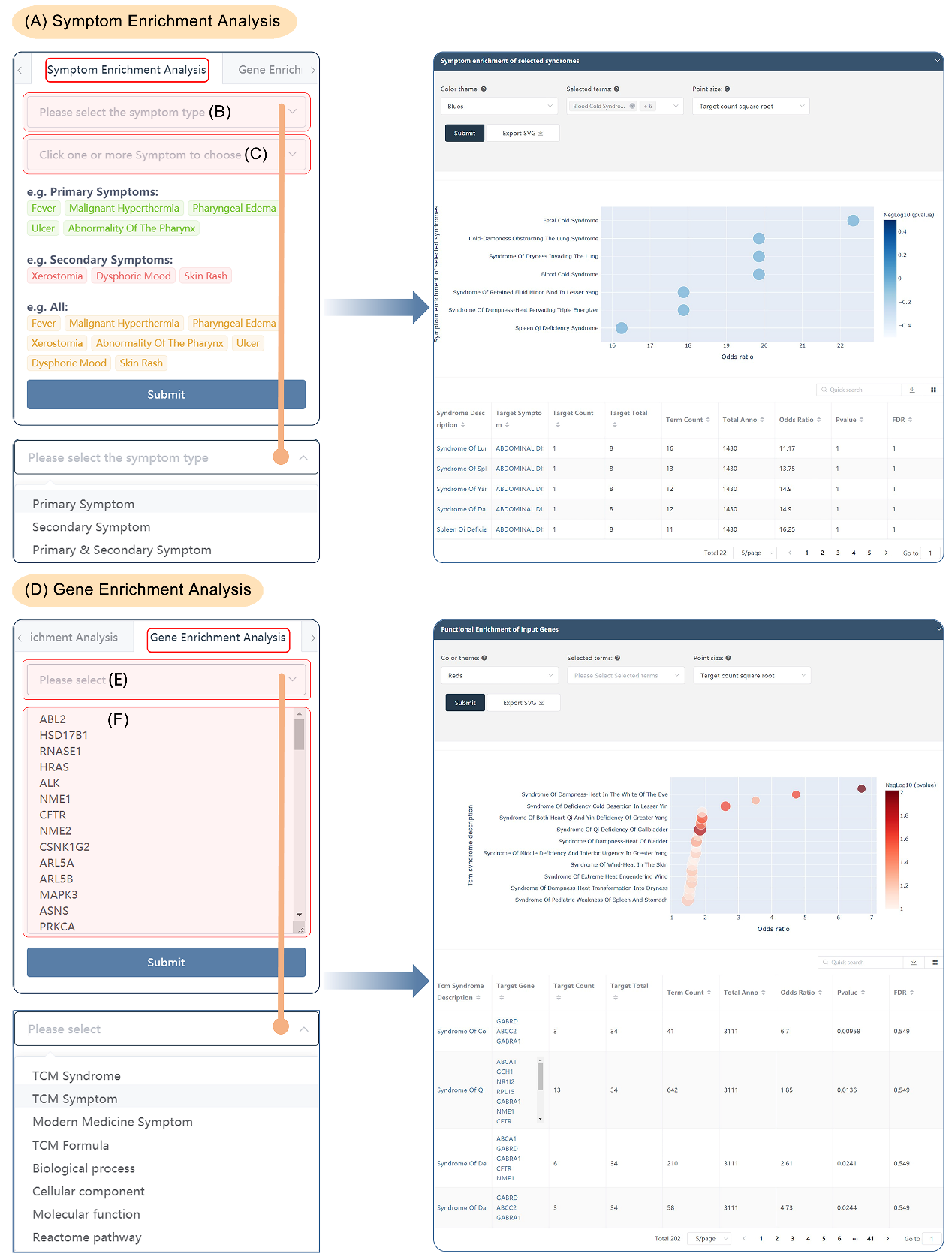
**

**FIGURE S1** Enrichment Analysis in the SoFDA platform.(A) Symptom enrichment analysis. (B) Symptom type. (C) Symptoms. (D) Gene enrichment analysis. (E) Enrichment type. (F) Gene list


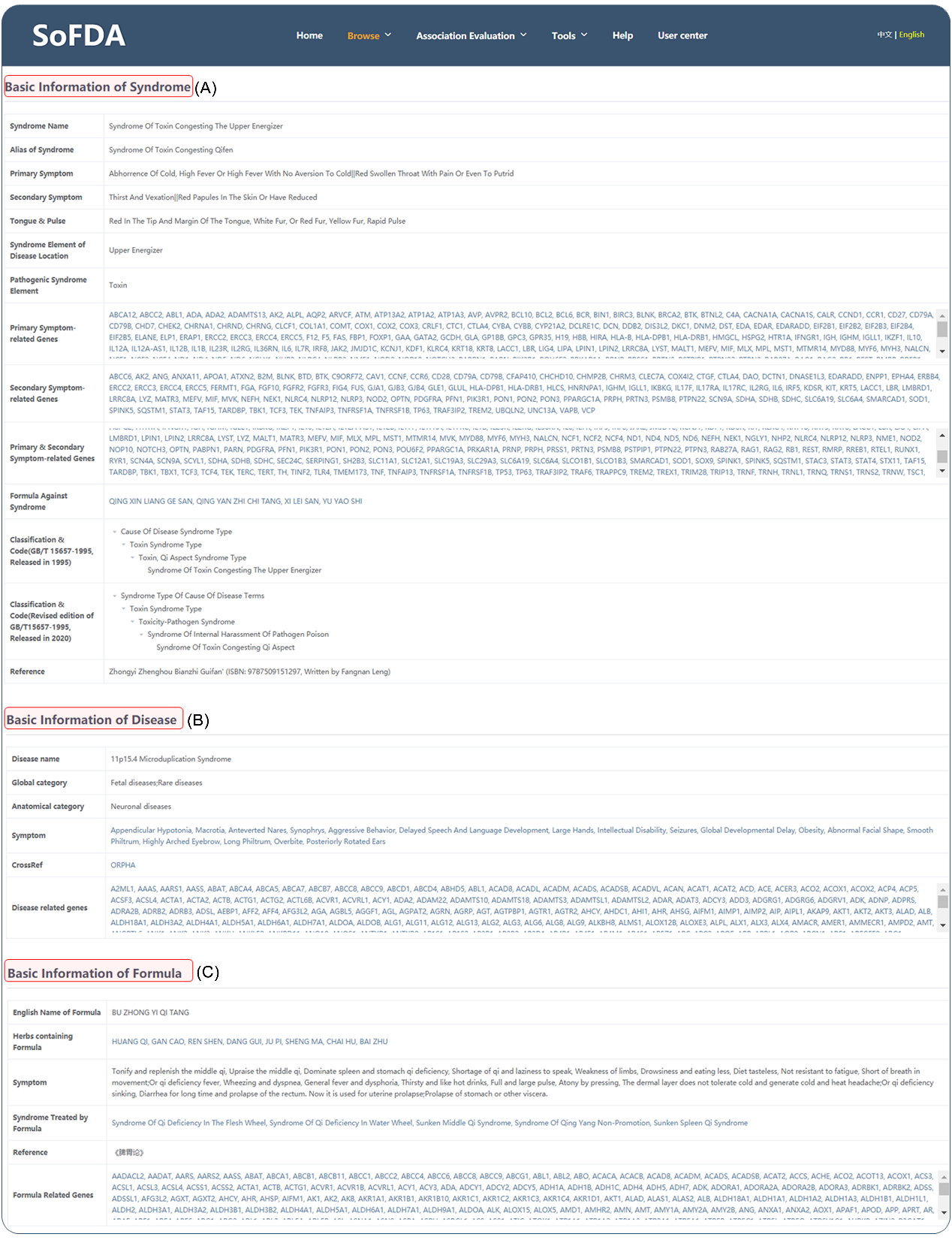


**FIGURE S2** Examples of the basic information of TCM syndromes, diseases and TCM formulas containing in the SoFDA platform (merge page). (A) TCM Syndrome. (B) Disease. (C) TCM Formulas


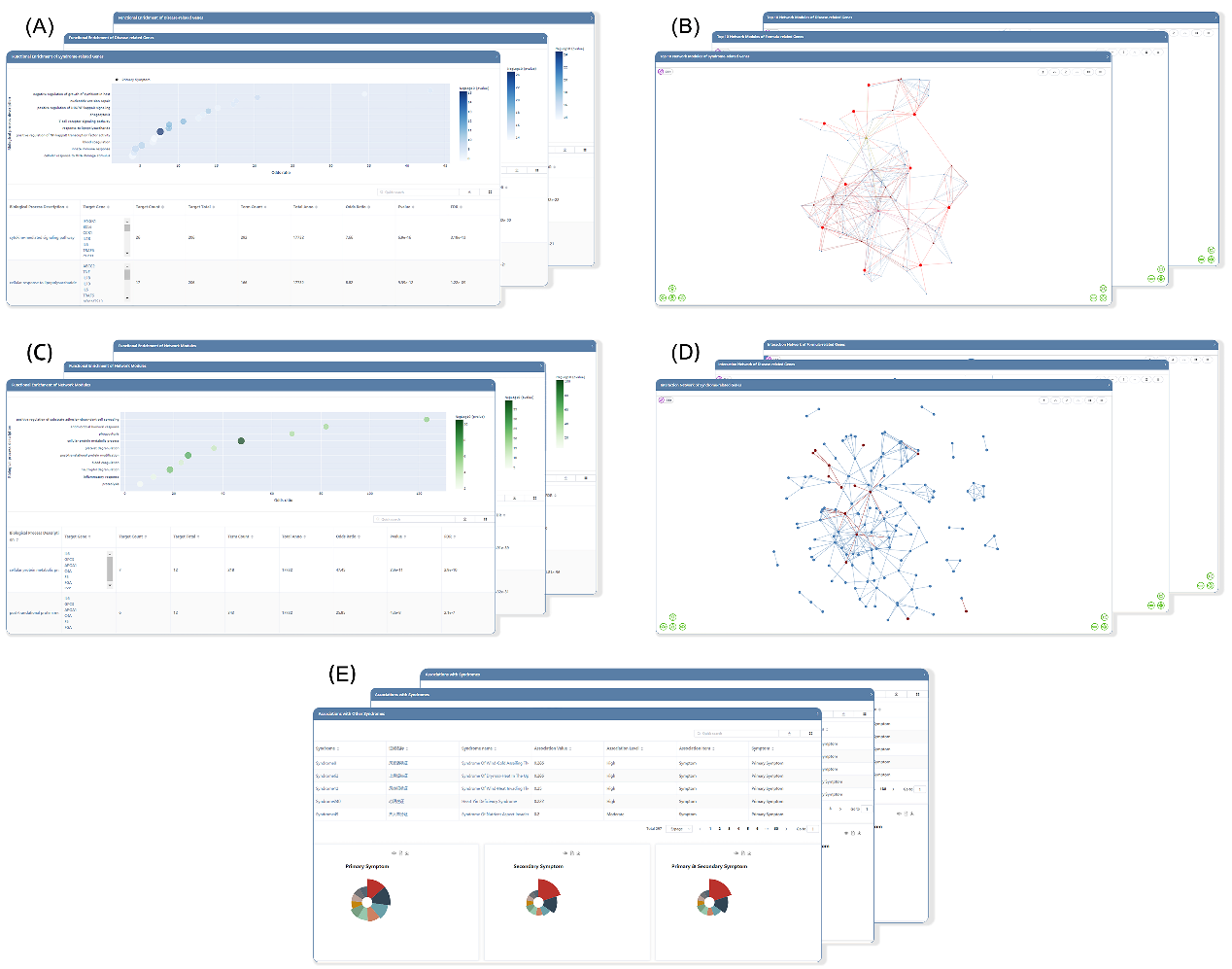


**FIGURE S3** Enrichment analysis and data visualization of the SoFDA platform. (A) Functional Enrichment of Syndrome/Disease/Formula-related Genes. (B) Interaction Network of Syndrome/Disease/Formula-related Genes. (C) Top 10 Network Modules of Syndrome/Disease/Formula-related Genes. (D) Functional Enrichment of Network Modules. (E) Associations with Other Syndromes


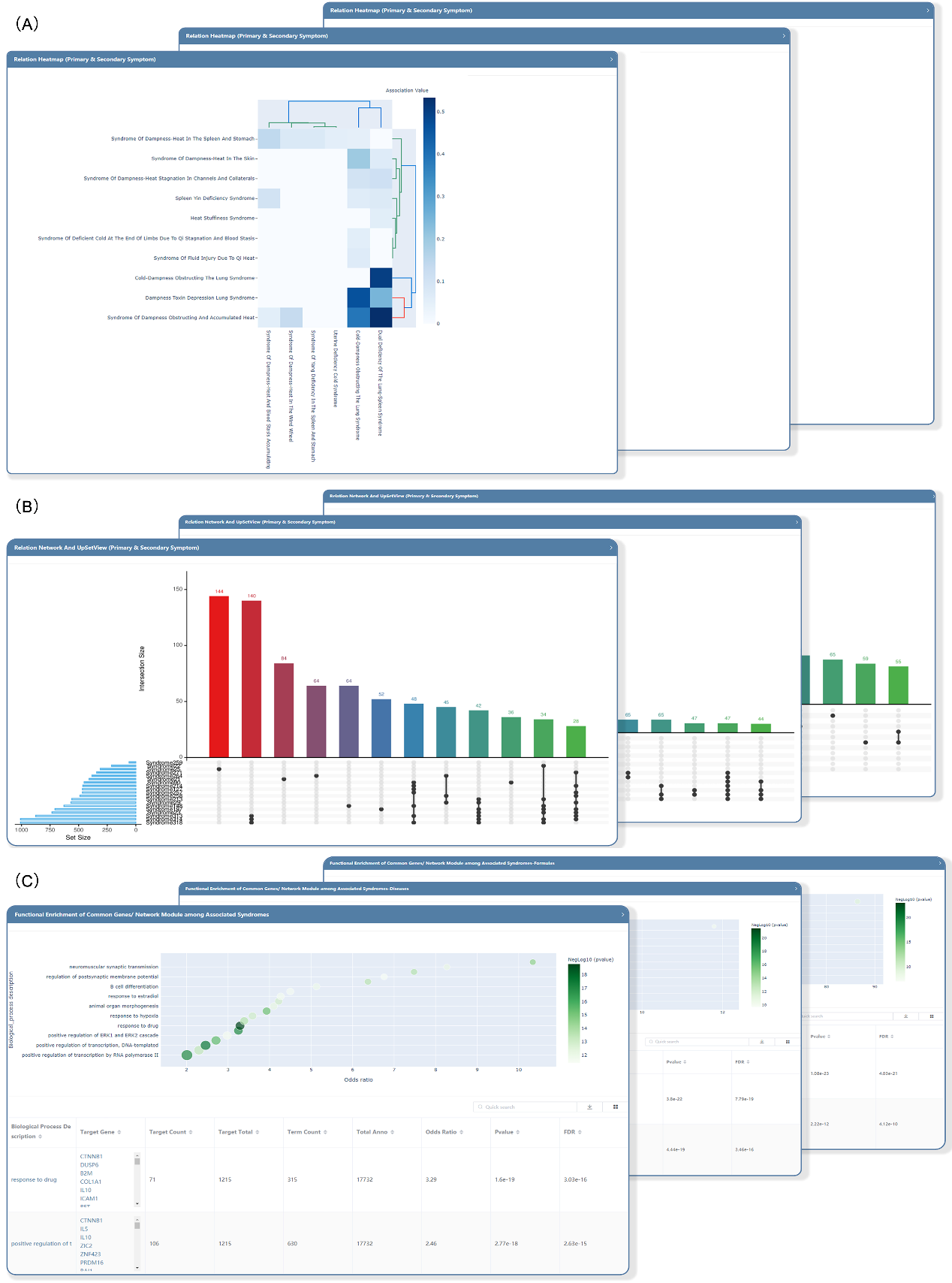


**FIGURE S4** Visualization of association evaluation results in the SoFDA platform. (A) The visualization of Syndrome-Syndrome Association. (B) The visualization of Disease-Syndrome Association. (C) The visualization of Formula-Syndrome Association


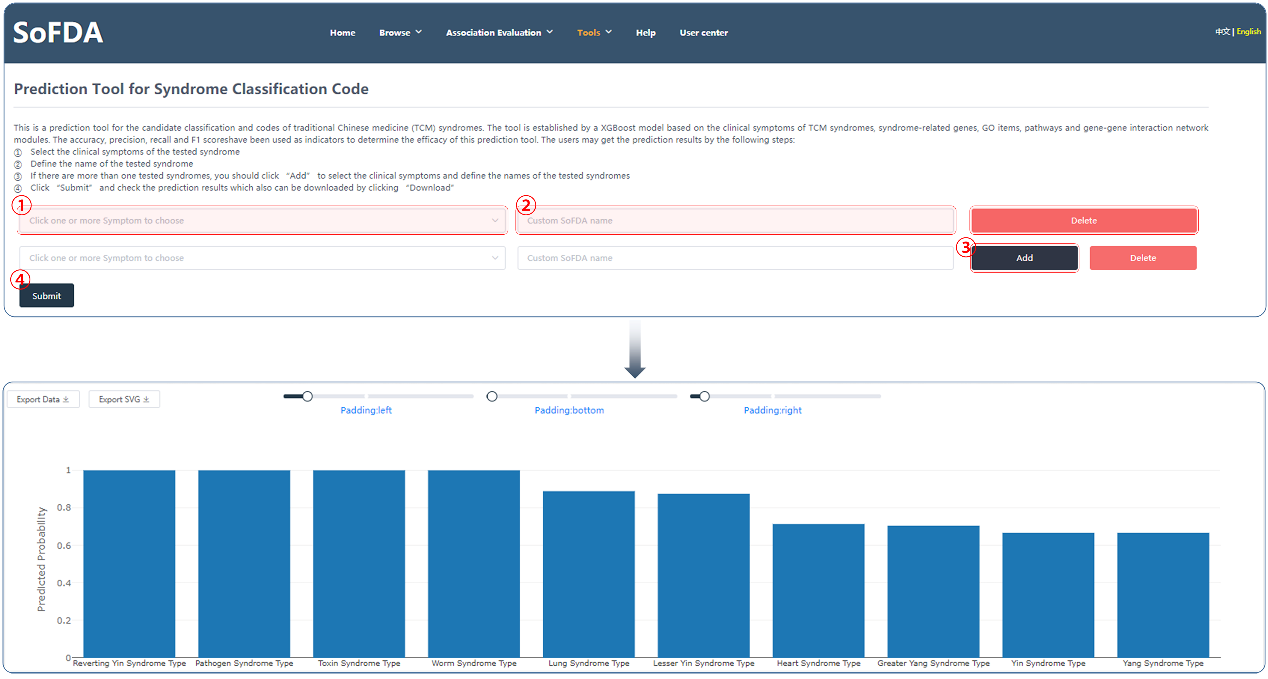


**FIGURE S5** The Prediction Tool for Syndrome Classification Code of SoFDA

**
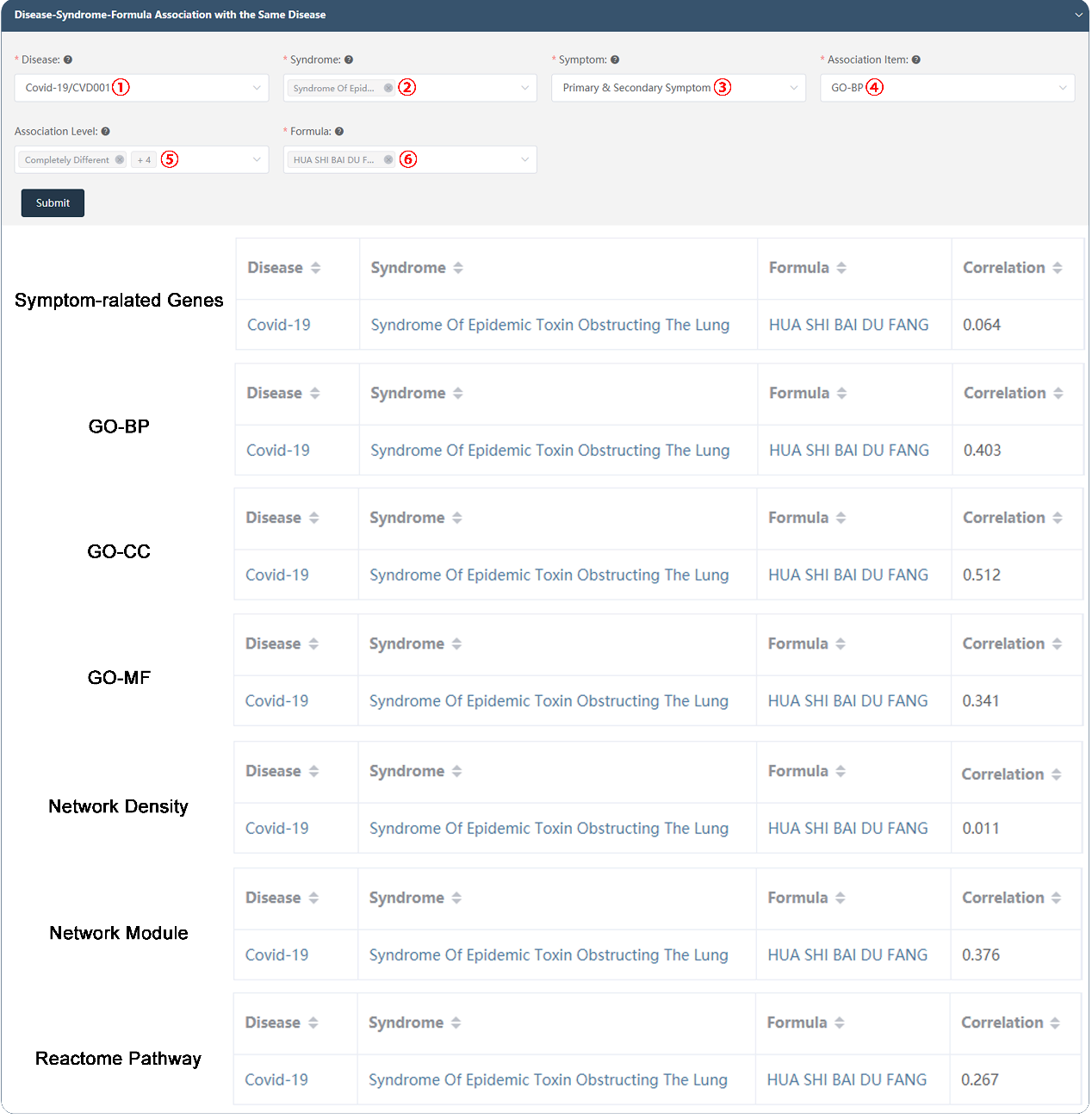
**

**FIGURE S6** Case study on COVID-19 of the SoFDA platform
